# The Work of a Revolutionary: A Psychobiography and Careerography of Angela Y. Davis

**DOI:** 10.5964/ejop.5507

**Published:** 2021-08-31

**Authors:** Jason D. Reynolds (Taewon Choi), Bridget M. Anton, Chiroshri Bhattacharjee, Megan E. Ingraham

**Affiliations:** 1 Department of Counseling Psychology, School of Education, University of San Francisco, San Francisco, CA, USA; 2 Department of Professional Psychology and Family Therapy, College of Education and Human Services, Seton Hall University, South Orange, NJ, USA; Nelson Mandela University, Port Elizabeth, South Africa

**Keywords:** Angela Y. Davis, psychobiography, careerography, social cognitive career theory, politicized collective identity

## Abstract

Dr. Angela Y. Davis is a political activist, academician, and writer who has navigated and discussed issues of race, class, gender, and USA social policies across her 75 years of life. Davis’s activism established her as the icon of a larger social movement and further related to her decision-making and legacy. Using psychobiographical methods, data were gathered through publicly available sources to explore Davis’s personal, professional, and representational life, as well as understand Davis’s lived experience through a socio-cultural-historical perspective. Two established theories, Social Cognitive Career Theory and Politicized Collective Identity model, were applied to Davis’s life. Findings suggested that in addition to her unique intersectional identities, a confluence of factors including growing up in a family of activists, incarceration, Federal Bureau of Investigation (FBI) surveillance, Communist Party involvement, marginalization within activist spaces, and practicing radical self-care impacted Davis committing to a life as an activist, academic, and the leader of a social movement.

*I’m no longer accepting the things I cannot change… I’m changing the things I cannot accept.*

— *Dr. Angela Y. Davis* (Peer, 2018)

Political activist, academician, writer, and African American revolutionist Dr. Angela Y. Davis became a master scholar and joined the USA Communist party in 1969. She was incarcerated for charges related to freeing political prisoners, during which time she wrote her first autobiography. Davis established herself as a revolutionary icon during the Civil Rights Movement. She has worked as a professor at various ranks at University of California Los Angeles (UCLA), the Claremont Colleges, San Francisco State University, and is currently Distinguished Professor Emerita at the University of California Santa Cruz (UCSC) where she continues her work as an activist fighting against discrimination and injustices. Davis has endured much pain and suffering throughout her life and professional career as a Black queer woman in her fight for social justice. She continues to inspire millions of people as a leading figurehead of a political movement through her dedication to advocacy and activism, and much can be learned from Davis’s life and the meaning she has ascribed to her life work.

Psychobiography, or psychological biography, is the intensive and purposeful study of an individual life through established psychological theories within a socio-cultural-historical context (Ponterotto & Reynolds, 2017; Schultz, 2005b). Psychobiography has been foundational to psychology, psychological theory development, and clinical interventions to better understand personhood and how to promote healing and well-being through the rigorous case study of an individual life (Ponterotto & Reynolds, 2017; Schultz, 2005b). Psychobiographers have shifted their focus by incorporating the impact career development and work experiences have on an individual’s life course, trajectory, and vocational contributions through careerography (Park-Taylor et al., 2021). The present study utilized Social Cognitive Career Theory (SCCT; Lent et al., 2002) as a lens for understanding Davis’s career decision-making in relation to her interests, values, and environment, and Simon and Klandermans’ (2001) Politicized Collective Identity (PCI) model allowed for deeper understanding of Davis’s contributions to social change. The authors discuss the initial psychobiographical and careerographical research questions, method, anchoring theories, then provide a sketch of Angela Davis’s life, and conclude with theory application and a discussion.

## Initial Research Questions

*Revolution is a serious thing, the most serious thing about a revolutionary’s life. When one commits oneself to the struggle, it must be for a lifetime.*

— *Dr. Angela Y. Davis *(Moore-Gilbert et al., 2014, p. 233)

This study is based in the application of two theories, SCCT and Simon and Klandermans’ (2001) PCI model in relation to the following research questions: 1) What factors, such as identity and historical and cultural experiences, are related to Davis’s career development? What role did self-efficacy play in her different career functions and goals? 2) How can Davis’s life be understood through the tenets of PCI, including historical antecedents, shared grievances with different political groups, and the involvement of the larger general public in her activism? 3) How does Davis’s work as an activist and scholar contribute to others’ development of PCI development?

## Method

### Procedure

The four-person research team initially researched psychobiography and psychobiographical methods. Next, the team researched and eventually chose Angela Davis as a subject because of her incredible contribution to society through writing, scholarship, and activism as a queer Black woman. A plethora of data on Davis’s life was available. The team chose Davis’s 1974 autobiography, books she had written, recorded interviews and commentary, and live conferences in which Davis was the keynote speaker over the course of a year. These sources were chosen because they provided a balanced range of media and were psychologically salient to understanding Davis across her full lifespan, with particular attention to her upbringing, young adult experiences including Communist Party involvement and incarceration, her intersectional identities, as well as her later life work accomplishments and activism (Schultz, 2005a). Next, the team began to construct a life sketch of Davis and explore possible psychological and social science theories to conceptualize Davis’s journey, with the goal of understanding Davis’s life and career success, as well as answering the research questions above.

#### Validity and Reliability

Issues of validity and reliability were considered. Attempts were made to triangulate data by using a variety of salient data sources (e.g., autobiography, books, recorded interviews, live in-person events and conferences). Davis was not, however, directly spoken to, nor were any friends or family members. While every psychobiography has some level of subjectivity and choosing psychological theories varies, attempts were made to provide balanced findings and a clear outline of how the project was completed.

#### Ethical Considerations

Prior to the project, the team had a limited understanding of Davis’s work. Ponterotto and Reynolds (2017) described the ethical challenges in choosing a subject to study and the potential temporal impacts of the project. The authors stated that writing about a living subject, compared to a person recently-deceased or long-deceased, has unique ethical challenges and concerns. Regardless of the subject chosen, Schultz (2005a) described the importance of exploring “why” this subject was chosen and how these reasons impact or may even interfere with each subsequent step in the psychobiographical research process, most importantly findings and interpretations. In choosing Davis, the team recognized that she is a living, breathing subject and that extremely high ethical vigilance and caution would be required in order to arrive at a balanced interpretation (Ponterotto & Reynolds, 2017). The team discussed the potential risk for distress or harm that could occur for Davis and her community from engaging in a psychobiographical study focused on her life accomplishments, career decisions, and social impacts. It was concluded that the potential impact would be extremely low, considering no sensitive or private health information was brought to light through the study (Ponterotto, 2015; Ponterotto & Reynolds, 2017).

### Anchoring Theories

The following two theories were used to gain a deeper understanding of Davis’s life, how she found her calling as an activist and educator, the meaning she ascribed to her work, and ways in which revolutionaries and activists may be better supported. To understand Davis’s life and mission, her psychobiography warrants a thorough review of her career life course. Since the central focus of this psychobiography and careerography is to highlight the many intersecting identities of the subject, the SCCT model was used to capture Davis’s dynamic life course. In addition, Simon and Klandermans’ (2001) PCI model was chosen to understand how Davis’s collective identity was influential in her work and accomplishments, and how her work may inspire future activists.

#### Social Cognitive Career Theory

While research focused on Black women in career and academic self-efficacy is limited, given its theoretical background SCCT appears suitable to guide Davis’s careerography. The model has two overarching elements that include cultural and ethnic dynamics such as early influences that serve as precursors of important socio-cognitive variables and ongoing contextual influences that serve as moderators, facilitators, or deterrents (Lent et al., 2002). The model also attends to individuals’ developmental stages and elaborates on how outcome beliefs are influenced by early and ongoing learning experiences; in addition, it focuses on how efficacy percepts and outcome expectations influence each other (Hackett & Byars, 1996).

SCCT captures domain-specific subjective well-being and is designed to honor unique cultural influences in the development of an individual’s career self-efficacy (Hackett & Byars, 1996). Byars-Winston and Rogers’ (2019) recent social cognitive model of academic and career choice explains ways in which dimensions of identity (e.g., race, ethnicity, gender, sexual orientation, socio-economic status) influence career efficacy beliefs, outcome expectations, interests, and goal setting. It is imperative to recognize the experiences of Black women in the USA during the time period when Davis was raised in the 1940s–1960s (i.e., the end of World War II and postwar Baby Boom; the Cold War; the Korean War; the Civil Rights Movement; Brown v. Board of Education ruling; the Bay of Pigs and Cuban Missile Crisis; the Vietnam War). SCCT also accounts for structural inequality, institutionalized discrimination, and other hurdles that minority groups often encounter (Byars-Winston & Rogers, 2019).

According to Bandura (1986), there are four major sources of efficacy information: performance accomplishments, vicarious learning, physiological and affective states, and verbal persuasion. Strong career self-efficacy expectations are linked to positive outcome expectations and facilitate performance attributions, such as increasing interest in areas in which one feels efficacious and promoting persistence in the face of obstacles (Lent et al., 2002). As outlined in the life sketch, Davis developed and strengthened her self-efficacy through her many activist accomplishments, her arrest and subsequent vindication, as well as her achievements as social activist, leader, professor, and queer Black woman in the 1960s and 1970s.

#### Politicized Collective Identity Model

Simon and Klandermans (2001) developed a social psychological model for PCI based around three triads. The first triad involves the reasons why individuals engage in PCI, including established collective identity, inter-group power struggles, and larger sociohistorical context. The second triad involves the antecedents to a PCI, including awareness of shared grievances, adversarial attributions, and societal involvement. The third triad conceptualizes PCI itself as the struggles between the ingroup and outgroup, with one usually representing the elite or authority, alongside the involvement of a larger third party such as the general public or a more powerful authority.

A collective identity is an individual’s identity shared with a group who have (or are perceived to have) some characteristic in common, such as race, ethnicity, gender, or political party (Deaux, 1996; Simon & Klandermans, 2001). Deaux (1996) described that collective identity is a subjective claim that relies on an individual’s personal acknowledgement of the identity as a self-defining piece. Therefore, in the PCI model, the third party witnessing the ingroup/outgroup conflicts may include individuals who share identities with those locked in the inter-group power struggles but have not claimed these identities as self-defining (Ashmore et al., 2004). In this model, PCI is a specialized form of collective identification committed to political consciousness and communal action, but not all collective identities require this type of engagement. At the individual level, several studies (Drury & Reicher, 2005; Klein et al., 2007; Stewart & McDermott, 2004; van Zomeren et al., 2008) identified that an individual’s endorsement of PCI transforms political projects into personally valuable labors, thereby creating activist or agentic identities based on individuals’ collective identities and connections to structural inequalities. Social change, Simon and Klandermans (2001) argued, occurs when subordinated groups develop a distinct collective identity, with associated goals, values, and beliefs, to contest dominant groups’ power as illegitimate.

Based on extant literature, the PCI model can be used to understand the experiences of Black women’s PCIs and meaning-making. For example, African American group consciousness and identification with feminist movements are significant predictors of political participation and willingness to undertake collective action (Dawson, 1994; Hercus, 1999; Tate, 1994). PCI can also offer a lens in considering Davis as not only an activist but as a symbol of her movements. Individuals can become emotionally attached to PCI groups as part of their self-concept, as well as develop affective ties to leaders and symbols, which further deepen their commitment to collective action (Ashmore et al., 2004). Therefore, PCI appears to be suitable for understanding both Davis’s own identities and motivations as well as her legacy as a symbol of these movements.

## A Life Sketch of Angela Davis

### Early Life

Dr. Angela Y. Davis was born on January 26, 1944, in Birmingham, Alabama. She lived in a segregated neighborhood and attended segregated schools in the 1940s and 1950s until the age of 15. Her neighborhood in Birmingham was labeled “Dynamite Hill” because her Black neighbors’ homes were targeted by the Ku Klux Klan (Davis, 1974, 2018). Both of Davis’s parents were teachers in segregated schools. Her mother earned a master’s degree during summer breaks from school, and shortly after the birth of Davis, her father quit teaching and became a mechanic (Davis, 1974; Weisman, 1972).

Davis is the oldest of four children: Fania Davis Jordan, Reginald Davis, and Benjamin Davis. Her parents were heavily involved in revolutionary and anti-racist work and were members of the National Association for the Advancement of Colored People (NAACP), which at the time was illegal in the eyes of the government, and the Southern Negro Youth Congress (SNYC), a group focused on creating alliances among Black people and defending the wrongfully accused (Davis, 1974; Platt, 2013; Radcliffe Institute for Advanced Study, 2019). Davis’s parents maintained close relationships with other revolutionaries in the neighborhood, including members of the underground Black Communist Party, with whom Davis spent ample time with as a child, likely influencing her future as an activist (CSPAN, 2009). From the age of six, Davis recalled her fear of the Federal Bureau of Investigation (FBI) as they closely monitored her family, due to her parents’ involvement with the Communist Party (CSPAN, 2009; Platt, 2013). Davis’s parents had a profound impact on the development of her beliefs and values, given her early exposure to revolutionary and anti-racist work through the NAACP and the Black Communist Party.

In 2018, Davis stated:

From the moment I became aware that Black people were treated as inferior, I can remember hearing my mother’s voice saying, ‘This is not the way things are supposed to be, this is not the way the world is supposed to be organized and one day they will be different’. (Davis, 2018, 36:07)

As a result, Davis learned how to imagine a world different from the world in which she lived. Davis acknowledged she walked down a path focused on activism and education that was carved out by her mother, even though she actively resisted this path as a child (CSPAN, 2009). Davis stated, “My own political activism is in many ways a legacy of my mother’s” (CSPAN, 2004, 3:52).

By 15, Davis became more aware of segregation in the South after several visits and spending a summer with family friends in New York City (Davis, 1974; Platt, 2013). These visits influenced Davis’ perception that freeing Black people in the south would free all Black people everywhere (Davis, 2019b). From a young age, Davis also recognized class differences among the Black community and often advocated for her marginalized schoolmates. In 1955, 11-year-old Davis was involved with an organization that held interracial discussion groups at a local church, which was subsequently burned as a result of the meetings (Platt, 2013). At age 14, Davis received a scholarship for Southern Black students to attend the Elizabeth Erwin High School, an integrated, progressive private school in New York City (Hornsby, 2011).

During her high school years, Davis became politically active and engaged with Communism and Marxist-Leninist literature and philosophy. In 1960, she joined Advance, a Marxist-Leninist youth group with connections to the Communist Party (Davis, 1974). After graduating from high school in 1961, Davis attended Brandeis University in Waltham, Massachusetts, during which she divided her time between activism and studying French literature and Western philosophy, including the Communist Manifesto (Davis, 2019a; Gay, 2011; Marx & Engels, 1848/2014; Platt, 2013). For Davis, exposure to Marxist theory allowed her to articulate the important changes that she felt society needed for improvement.

### Emerging and Young Adulthood

Davis continued her education in graduate school at the Frankfurt School in West Germany and joined the anti-war organization Students for a Democratic Society (SDS), protesting the Vietnam War at the USA Embassy (Davis, 1974). She studied under Herbert Marcuse, a political philosopher, social critic, and supporter of the New Left and the protest movements in the 1960s (Cobb & Abromeit, 2004). According to Davis, Herbert Marcuse taught her that it was possible to be an academic and an activist, a scholar and a revolutionary (Juutilainen, 1996). In September 1963, Davis was notified of the 16th Street Baptist Church bombing in Birmingham, resulting in the death of four girls with whom Davis was close friends (Davis, 1974). This horrific news, combined with the growing Black Liberation Movement in the USA, led Davis to transfer from the Frankfurt School to the University of California San Diego to continue her academics alongside her involvement with the movement, before completing her Ph.D. in East Germany at Humboldt University of Berlin (Davis, 1974).

The beginning of Davis’s commitment to the freedom of political prisoners was seen through her work as the head of Bobby Seale’s defense committee in Los Angeles (Davis, 2016). In 1969, Davis joined the Black Panther Party (BPP) and the Los Angeles Student Nonviolent Coordinating Committee (SNCC) while she was teaching in the Philosophy Department at the University of California in Los Angeles (UCLA). Notably, Davis recognized issues in the BPP and SNCC regarding women’s roles and felt compelled to join intersectional organizations that addressed class as well as race and gender, such as the Communist Party, the National Alliance Against Racism and Political Repression, and the Black Women’s Political Caucus (Platt, 2013).

During this time, Davis illegally traveled to Cuba to protest the USA trade blockade (Davis, 1974).

On June 19, 1970, Davis was fired from UCLA due to her association with the Communist Party and using inflammatory language (Weisman, 1972). On August 7, 1970, Jonathan Jackson opened fire in a San Rafael courtroom; the aftermath of this conflict resulted in the deaths of Judge Harold J. Haley, James D. McClain, and William A. Christmas (Caldwell, 1970). Unknown to Davis, Jonathan Jackson used guns which were registered in her name, and subsequently, Davis became a fugitive. On August 19, 1970, Davis was added to the FBI’s Most-Wanted List. On October 13, 1970, Davis was charged with murder, kidnapping, and conspiracy (Davis, 1974). During her initial days of detention, Davis’s friends and family provided support and encouragement when she lost sight of her goals. These remarks resembled the consistent theme of Davis’s journey as a radical revolutionist: collective movement.

In an interview, Davis stated:

I think I was able to use my time in jail productively…there was an enormous movement on the outside. I had family, I had friends, I had colleagues and comrades all over the world who were concerned about my predicament. So, I didn’t feel alone in solitary confinement. (CSPAN, 2009, 1:01:30)

Uniquely, Davis learned yoga and practiced karate in jail. However, she was emotionally and psychologically impacted by her experience in solitary confinement. Davis spent 13 weeks on trial, 16 months in jail, and a million dollars on court fees before being acquitted 11-1 of all charges, all of which would have a lasting impact on her life’s work (Hager, 1972).

Davis wrote her first autobiography at the age of 28 (Davis, 1974). This work serves as a chronicle of the incidents of late 1960s and early 1970s through the lens of a young Black female activist and a member of the Communist party fighting for liberation. Throughout her autobiography, Davis reminded the readers that her fame was merely luck and stated that any other political prisoner could have been subjected to such extraordinary life events. Davis described in meticulous detail her time in prison, the organized rallies, and the court trials through her memoir (Davis, 1974). She described the jury, the racist comments, the internal struggles, the identity transformation, and the evolving value system through powerful and persuasive language painting a very prominent picture for the reader (Davis, 1974).

### Middle Adulthood

Following Davis’s acquittal, she taught at various institutions (San Francisco State University, UCSC) in various departments (Women’s Studies, Philosophy, Interdisciplinary Ph.D. program History of Consciousness). She was a Full Professor at UCSC in the History of Consciousness and Feminist Studies Department for 15 years until her retirement in 2008 where she remains a Distinguished Professor Emerita (Rappaport, 2019). Since its creation in 1972, Davis has co-chaired the National Alliance Against Racist and Political Repression, with which she has led demonstrations in 48 states (Beyette, 1989). In the early 1980s, Davis organized a demonstration in Montgomery, Alabama to raise awareness for Johnny Harris, a Black man on death row for a conviction of murder and rape (Beyette, 1989).

In 1980, Davis married Hilton Braithwaite, a photographer and faculty colleague at San Francisco State University, but their relationship ended in divorce in 1987 (Beyette, 1989; People Staff, 1980). In 1997, Davis came out publicly as lesbian at the Diverse Sexuality and Gender Alliance at Johns Hopkins University (Gay, 2011). Notably, Davis stated that she preferred romantic relationships remain private (Miles, 1998).

### Late Adulthood

Davis’s mother was diagnosed with Alzheimer’s disease (CSPAN, 2004, 4:05) and passed away in 2007, one of the major sources of inspiration for Davis in her anti-racist and activism work. In her late sixties, Davis became heavily involved with solidarity activism in Palestine. In 2011, she traveled to Palestine with a group of Black, Indigenous, and People of Color (BIPOC) feminist scholars and activists and formed a campaign for Israel to end modern-day racial segregation (Chokshi, 2019; Barat, 2014). In 2019, Birmingham Civil Rights Institute rescinded its highest honor, the Fred Shuttlesworth Human Rights Award, from Davis due to this activism for Palestine (Chokshi, 2019).

Presently, Davis continues her committed work for social justice. Since her release from prison in 1972, she has written over a dozen books and continues to speak at invited talks and symposiums for academic and lay communities (History, 2009)*.* Davis reported she is currently in the process of writing another book on prisons and American history (Davis, 2020a). She is on the executive board of the Women of Color Resource Center (WCRC) in the San Francisco Bay area (Davis, 2020a) and also works with Justice Now which is an organization that provides legal assistance to women in prisons and advocates for prison abolition (Davis, 2020a). Davis continues to speak at local colleges and events to continue the discussion on prison abolition, radical feminism, LGBTQIA+ rights, and racial and social justice. During the current coronavirus disease 2019 (COVID-19) pandemic, Davis advocated for prison abolition by spreading the news of the San Quentin prison social-distancing protest and the social inequalities exemplified with limited healthcare access for people of color as highlighted during this pandemic (Davis, 2020a).

Presently, Davis practices daily yoga, Pilates, meditation, (Davis, 2020a) and veganism (Davis, 2019b). Her dedication to her overall wellbeing exemplifies that staying healthy allows her to continue her dedicated work for many more years to come. Recently, Davis spoke about her radical self-care practices, specifically during pandemic times, with an organization for Black women’s health called GirlTrek (Davis, 2020b). Davis defined radical self-care as “self-care that involves community as well, self-care that is not simply individualistic…self-care that helps us all to heal” (Davis, 2020b, 44:18).

## Theory Application

Dr. Angela Y. Davis’s life was explored using SCCT and PCI models in an effort to gain additional insight and understanding to how Davis’s identity and the meaning she ascribed to her lived experiences as a queer Black woman may have impacted her career as an academic, activist, and revolutionist, as well as how her work led her to developing a PCI and subsequently became a symbol for her movements.

### Social Cognitive Career Theory

Contemporary society is increasingly shaped by transnational interdependencies; what happens economically and politically in one nation can have geopolitical impacts across the globe. One aspect of SCCT is to understand the collective efficacy in changing societies (Bandura, 1999). Davis’s activism called upon global interconnectedness, leading activists from around the world to collectively empower the marginalized and the vulnerable. Her work not only expanded across the world but also challenged the efficacy of governmental systems. She openly opposed structural racism and sexism, the prison-industrial complex, the broken immigration system, capitalism, and imperialism (Davis, 1974). Just as SCCT predicts, “bureaucracies thwart effective social action” (Bandura, 1999, p. 68). Davis faced criticism, threats, imprisonment, rejection, and disappointment, yet continued to pursue her life’s work as a revolutionist and academic. How was she able to maintain her collective action despite these obstacles? An explanation for her resiliency may be her ability for self-development, continual self-renewal, and self-regulatory efficacy (Bandura, 1999). According to Bandura (1999), one way to regulate self-efficacy is to gain primacy in diverse spheres of life. In Davis’s case it could be her educational domain, occupational pursuits, close interpersonal relationships, and conception of health and well-being.

Davis’s developmental trajectory, unique cultural experiences, career choices, self-efficacy, strengths, and struggles can be understood by the tenets of SCCT. In accordance with SCCT, Davis’s personal and professional goals are consistent with the views of her personal capabilities and outcomes she expected to attain. From her interviews and writings, we witnessed how her childhood, family system, educational upbringing, and parental involvement in social justice movements acted as precursors of important socio-cognitive variables, strengthening Davis’s self-efficacy. Her parents modelled how to stand up against injustice which left her with a sense of duty to support her community (Davis, 1974). Davis recognized her role as an activist very early on in life; she embraced it by addressing the issues of institutional injustice by making her work a collective endeavor rather than a personal agenda which gave her a sense of meaning, purpose, and motivation that went beyond her personal goals (CSPAN, 2004).

As an author, all of Davis’s works have encompassed her salient identities. Her autobiography highlighted her own powerful story which she narrated with much humor and vigor, giving readers a glimpse of her perseverance and conviction. She followed her passion and continued to write books that purposefully demonstrated the intersectionality of her multiple marginalized and privileged identities (Davis, 2018). Her narrative evolved throughout the years, staying congruent with the time and her developmental stage. The books she wrote demonstrated her insatiable desire to see radical changes for a more socially-just and egalitarian world, just as her mother had imagined. Davis’s writing illuminated the struggles of the oppressed, connecting the audience cognitively and emotionally.

As SCCT predicts, Davis’s variant roles as a writer, scholar, Black feminist, activist, professor, and woman of color each drove the same message which strengthened her self-efficacy and brought congruency to the meaning of her work and her sense of self. Even through difficult times as a political prisoner, she found a purpose. Her critical understanding of the systematic oppression, social power, and public concerns about racism evoked the type of motivation she needed to persevere as a leader (Byars-Winston & Rogers, 2019). Just as she did as an author and activist, she continued to elevate society’s political, racial, and social consciousness as a professor and speaker in diverse cultural spaces and platforms.

### Politicized Collective Identity Model

*I was unwilling to render my life as a personal “adventure”—as though there were a “real” person separate and apart from the political person.*

— *Dr. Angela Y. Davis* (Davis, 1974)

Davis’s life and meaning-making can be understood using the PCI model’s three triads. In the first triad, reasons for engagement in PCI, Davis was familiar with inter-group power struggles from childhood. Racially motivated bombings of Southern neighborhoods and churches by White Supremacist groups, segregation, and the FBI’s surveillance of her family made Davis aware of the ways in which inequality was systematically maintained (CSPAN, 2009; Davis, 1974, 2018; Platt, 2013; Weisman, 1972). Davis’s experiences with racialized violence and remembrance of her parents speaking out against oppression both personally and through sociopolitical organizations laid the groundwork for the politicization of Davis’s collective identity.

In the second triad, antecedents to PCI, Davis’ awareness of shared grievances are apparent through her involvement with several different organizations across her life, including anti-war, Communism, Black liberation, interracial discussion, and anti-incarceration groups (Davis, 1974; Hornsby, 2011; Platt, 2013). With this range of involvement, Davis utilized an intersectional lens regarding shared grievances, speaking out not only against injustices based on race but also based on gender, class, and religion (Davis, 1981, 1989; Platt, 2013). This wider lens capturing the shared grievances of the larger population encouraged the triangulation of society in these inter-group struggles.

The third triad understands PCI as the struggle between the ingroup and outgroup with the involvement of a larger third party, such as the general public or a more powerful authority. With Davis’s widespread activism, this ingroup/outgroup dynamic can change. Examples of the ingroup include the FBI, White Supremacist organizations, the prison-industrial complex, and beneficiaries of unchecked capitalism, to name a few. Both the ingroup and outgroup contribute to this involvement of the larger society. The FBI contributed, for example, by placing Davis on the FBI’s Most Wanted List and thus bringing her motivations to the national stage (Davis, 1974). The media contributed through publicizing her manhunt, trial, and continued activism (Davis, 1974).

Similarly, Davis maintains her involvement with the larger society and the development of others’ PCI through her educational and activist roles. As an educator in topics such as the history of consciousness, philosophy, and feminist studies, she utilizes her platform to teach these same concepts of collective identity, involvement in sociopolitical movements, and radical self-care. As a writer, she speaks about her own experiences and meaning-making, thereby challenging the powerful ingroup’s narrative of the events, and writes about global power struggles to educate the public. As an activist, she continues to demonstrate a wide, intersectional lens which facilitates others to find commonality and create meaning in their shared grievances. By raising awareness about power struggles, allowing herself to be identified as an icon and symbol of massive sociopolitical movements, and educating others about the power in collective identity and communal action, Davis’s legacy continues to foster the development of others’ three PCI triads (Simon & Klandermans, 2001).

In relation to the development of an individual’s PCI, Davis recently acknowledged in a keynote address that one can feel exhausted in the fight for social justice, and recommended that people take time to rest in order to continue doing long-term activist work (Davis, 2019a). She suggested cultivating community and not doing this work alone so when you do step back for some rest, someone else will step forward to share the responsibility. Davis also suggested that activists acknowledge the challenges and trauma associated with doing this work and incorporate restful and healing practices into daily routines such as meditation and yoga. Davis stated,

Have fun. We are not going to move in a forward direction unless we can feel pleasure in the course of engaging in the struggle. If it’s all sacrifice, if it’s, you know all bad, then no. But we have to learn how to generate joy and pleasure in the process of doing our organizing. And I think that’s what has allowed me to remain in this movement. (Davis, 2019b, 1:32:36)

Davis emphasized the importance of having a strong support system that ensures a sense of community especially for people from minority backgrounds. She mentioned the term “radical self-care” in one of her interviews with GirlTrek’s #daughterof campaign, where she explained the value of self-care that involves the community. She shared,

When I was in jail, it was a pretty frightening experience. But I began to realize at one point when I started receiving letters from people who were active in the Black community, open letter from James Baldwin, people in Africa, Asia, Europe, and Latin America coming together to fight for my freedom. I realized that the source of so much of my fear was that I felt alone. The moment I realized that I was not alone, it wasn’t that I seized feeling afraid, but the emotions of gratitude towards those that were generating solidarity became so vast that those emotions overwhelmed the sense of fear. (Davis, 2020b, 46:00–48:04)

She reminded her audience that the idea of self-care is often very individualistic in nature that focuses on helping oneself. However, social justice work does not happen individually. Davis developed into a strong, independent woman, while simultaneously learned to rely on her community in order to remain steadfast in the struggle for justice, equality and freedom (Dixon & Garrison, 2020). Radical self-care, according to Davis, requires us to involve the entire community which can further enhance the healing process through a collective identity. Activism is most meaningful and effective when performed individually, collectively, and systemically. Radical self-care techniques, particularly in response to adverse and traumatic experiences in life, may also include personal counseling and therapy to process trauma and the ongoing struggles in the fight for justice.

## Discussion

Davis’s early childhood experiences and pedigree as a child of activists might suggest she was destined to become a famous Black female revolutionist. Yet to become a leader and the face of a world-wide social movement requires more than simply heredity and early exposure. Davis’s experiences with hardship, in particular her incarceration in her late twenties, her commitment to the Communist party and the subsequent discrimination she experienced from the USA government, and her experiences as a Black woman (later coming out as lesbian) in largely male-driven activist spaces had a profound impact on her work as a revolutionist and affected the causes to which she dedicated her time and energy. It was never simply about fighting for racial justice, although race remained a central focus of her work, but rather her work more broadly attended to the intersections of multiple disenfranchised identities such as class and gender. She also fought for other social issues such as prison reform, the freeing of political prisoners, the Israeli-Palestinian conflict, and protesting the Vietnam War, which provided her with a wider panorama than some of the other contemporary revolutionists. Davis’s focus on these intersections was ahead of her time in many ways, given that third-wave feminism and intersectionality movements did not officially emerge until decades later (Crenshaw, 1989).

By conceptualizing Davis’s life through both the SCCT and PCI theories, we gained a deeper understanding of the important life experiences that unlocked her true potential for activism and leading a social movement. Through her status as an outgroup member of multiple disenfranchised communities, including her outgroup status as a woman in male-dominated activist spaces, as a Black woman in White feminist-dominated spaces, and as an anti-incarceration, Black Liberation, and Communist Party member and leader in FBI- and White Supremacist-led spaces and systems, Davis was a driving force behind intersectional work including Black women’s liberation, the prison-industrial complex, and international solidarity. Through her continued activist and revolutionist work, Davis continues to be an inspiring teacher and mentor for all. Davis’s life was full of hardship and trauma as a Black queer woman, yet her determination, passion, and unwavering dedication to the movement are what kept her on track to become one of history’s most revered and celebrated female social activists and revolutionists. Her continued work as an activist and educator within a politicized collective movement speaks to her commitment, durability, and continuous leadership abilities, as well as her ability to practice self-care and find support in community building. The findings and interpretations from this paper only begin to elucidate the full complexity of Davis’s remarkable life and journey.

Davis’s lifework to create a more just society for all is needed now more than ever. The COVID-19 pandemic has shed light on the impact of centuries of systemic and institutional racism that has left BIPOC communities at significantly higher risk for alarming health disparities, as well as the deleterious impact it has had on women (Cheng et al., 2021). These same BIPOC communities continue to be killed both at the hands of police officers and White Supremacists in 2021. Thus, Davis’s messages are more relevant than ever. Much work is required, and it must be done collectively through radical self-care, restorative justice, and community healing.
